# Novel Three-Dimensional Bladder Reconstruction Model from B-Mode Ultrasound Image to Improve the Accuracy of Bladder Volume Measurement

**DOI:** 10.3390/s21144893

**Published:** 2021-07-18

**Authors:** Meng-Lin Chang, Hsiao-Chi Li, Chang-Keng Liu, Han-Sun Chiang, Chien-Chang Hsu

**Affiliations:** 1 Department of Urology, Fu Jen Catholic University Hospital, Fu Jen Catholic University, New Taipei 24352, Taiwan; oro.tidyscoundrel@gmail.com (M.-L.C.); 053824@mail.fju.edu.tw (H.-S.C.); 2 School of Medicine, College of Medicine, Fu Jen Catholic University, New Taipei 242062, Taiwan; 3 Graduate Institute of Applied Science and Engineering, Fu Jen Catholic University, New Taipei 242062, Taiwan; 4 Department of Computer Science and Information Engineering, Fu Jen Catholic University, New Taipei 242062, Taiwan; hcli@csie.fju.edu.tw; 5 Graduate Institute of Computer Science and Information Engineering, Fu Jen Catholic University, New Taipei 242062, Taiwan; h152074716@gmail.com; 6 Division of Urology, Department of Surgery, Cardinal Tien Hospital, New Taipei 23148, Taiwan

**Keywords:** imaging, algorithms, bladder, post-void residual urine (PVR), computer-assisted image processing, ultrasound

## Abstract

Traditional bladder volume measurement from B-mode (two-dimensional) ultrasound has been found to produce inaccurate results, and thus in this work we aim to improve the accuracy of measurement from B-mode ultrasound. A total of 75 electronic medical records including ultrasonic images were reviewed retrospectively from 64 patients. We put forward a novel bladder volume measurement method, in which a three-dimensional (3D) reconstruction model was established from conventional two-dimensional (2D) ultrasonic images to estimate the bladder volume. The differences and relationships were analyzed among the actual volume, the traditional estimated volume, and the new reconstruction model estimated volume. We also compared the data in different volume groups from small volume to high volume. The mean actual volume is 531.8 mL and the standard deviation is 268.7 mL; the mean percentage error of traditional estimation is −28%. In our new bladder measurement method, the mean percentage error is −10.18% (N = 2), −4.72% (N = 3), −0.33% (N = 4), and 2.58% (N = 5). There is no significant difference between the actual volume and our new bladder measurement method (N = 4) in all data or the divided four groups. The estimated volumes from the traditional method or our new method are highly correlated with the actual volume. Our data show that the three-dimensional bladder reconstruction model provides an accurate measurement from conventional B-mode ultrasonic images compared with the traditional method. The accuracy is seen across different groups of volume, and thus we can conclude that this is a reliable and economical volume measurement model that can be applied in general software or in apps on mobile devices.

## 1. Introduction

The volume of urine inside the bladder is essential in the thinking process of differential diagnosis in lower urinary tract diseases. Storing and emptying the urine are the main two functions of the bladder. The bladder normally stores urine gradually, and the sensation of urgency happens when reaching the maximal capacity of the bladder, at which point people would endure the urgency feeling and then empty their bladder under self-control. Any abnormal change during the storing and emptying phase is considered to be lower urinary tract disease. Incontinence is the involuntary emptying of the bladder that happens during the storing phase. Bladder outlet obstruction is a blockage in the way of the urine flow during the emptying phase. Many female patients suffering from urgency and urge incontinence due to an overactive bladder are found to have a minimal amount of residual urine. On the other hand, patients who have urinary retention with a large amount of residual urine also suffer from frequency and urgency because of incomplete bladder emptying. The condition might eventually deteriorate into overflow incontinence. In clinical practices, the measurement of bladder urine plays an important role when managing many uro-gynecological disorders in females and lower urinary tract diseases in both females and males.

Historically, data for measuring urine amount in the bladder were first obtained and calculated by certain invasive procedures, such as urinary catheterization or trans-abdominal puncture on the distended bladder. In addition to the physical discomforts which the patients have to endure, there are risks of complications, such as urethral injury and variable degrees of urinary tract infection [1]. Holmes J. was the pioneer to use ultrasonic studies of the urinary bladder in 1967 [2]. Since then, using non-invasive ultrasound to assess bladder volume became more and more popular. With this method, the bladder volume is measured using an ellipsoid or ellipsoid-like equation model from the transverse and sagittal view of the bladder ultrasound image [2,3]. However, inaccurate measurement was reported with a mean error of 11.5–35%, and irregular bladder shape can cause dramatic differences between the estimation and actual bladder volume [4]. Three-dimensional ultrasound is then developed and used to estimate bladder volume with better accuracy of 87–95% than 2D conventional ultrasound [5,6]. Nagle et al. compared 2D ultrasound with two estimated methods to 3D ultrasound in the measurement of the bladder volume. They found that the modified traditional bladder volume estimation from 2D ultrasound may increase accuracy and minimize the difference with 3D ultrasound measurement [7]. Different ultrasound devices may be another factor that affects the accuracy of bladder volume measurement. Two different commercial 3D ultrasound devices were validated by Brouwer et al., and the validation showed an overestimation of +17.5% in one device, compared to an underestimation of −4.1 to −6.3% in another device [8].

Some works of bladder volume focus on the automatic detection of bladder ultrasonic images. Traditional bladder volume estimation is calculated by manually selecting the tri-axis of the bladder; Matsumoto et al. used an artificial intelligence technique to perform the auto-detection of three diameters of the bladder to save time and to make detection more convenient [9]. The advanced fuzzy edge detector was developed to improve the selection of images where the border is blurred [10,11]. Fuzzy edge detection is also applied in medical image processing to resolve the vagueness that is usually found on the edge of medical images, such as retinal blood vessel detection [12] or musculoskeletal ultrasound images [13].

Although three-dimensional ultrasound has better accuracy in bladder volume estimation, three-dimensional ultrasound is not as popular as 2D conventional ultrasound and is not usually available in the general department in the hospital. In this study, we develop a novel bladder volume measurement method of 3D reconstruction model from B-mode (2D) conventional ultrasound images to increase the accuracy of bladder volume measurement.

## 2. Materials and Methods

**Figure 1:**
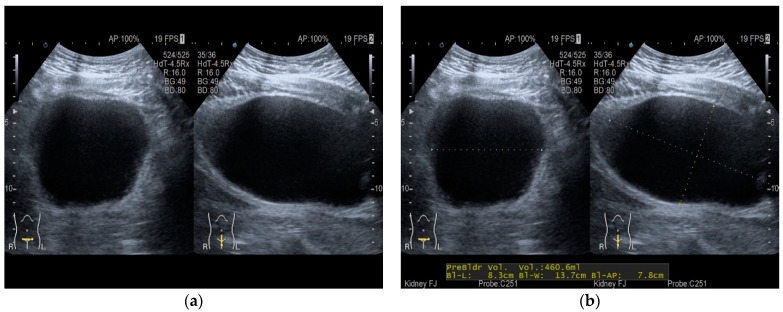
Traditional bladder volume estimation. (**a**) B-mode ultrasound of bladder, with transverse view (**left**) and sagittal view (**right**) shown. (**b**) Width is measured from a transverse view (**left**); height and length are measured from a sagittal view (**right**).

In this work, there were 75 bladder sonographic images from 64 patients with lower urinary tract dysfunction; these were collected retrospectively from medical records. Of these, 4 patients have good self-voiding with ultrasound confirming no residual urine inside the bladder; 11 patients have incomplete bladder emptying and each have two bladder ultrasonic images, i.e., pre-voiding and post-voiding. The other 49 patients have urine retention, and catheterization was performed. The ultrasound was performed trans-abdominally with the B-mode (2D) convex probe. The maximal bladder volume images were obtained in transverse view and sagittal view. Then, the maximal width was measured in the transverse view. The maximal height and length were measured in the sagittal view. The bladder volume was estimated using the traditional bladder volume measurement method (Figure 1), which considers the bladder as an ellipsoid with the following formula [14]:

(1) $$\begin{array}{l} Bladder\;Volume\;\left(Ellipsoid\right)=\left(\frac{Width}{2}\right)\times \left(\frac{Height}{2}\right)\times \left(\frac{Length}{2}\right)\times \frac{4}{3}\pi \\ =Width\times Height\times Length\times 0.52 \end{array}$$

We used a superellipse shape modification in our 3D reconstruction model. Superellipse was developed by French mathematician Gabriel Lamé in the 19th century [15]. The superellipse is defined as the following equation:

(2) $${\left|\frac{x}{a}\right|}^{n}+{\left|\frac{y}{b}\right|}^{n}=1\;\;\;\;\;=>\;\;\;\;\;\left|\frac{y}{b}\right|=\sqrt[{n}]{1-{\left|\frac{x}{a}\right|}^{n}}$$

**Figure 2:**
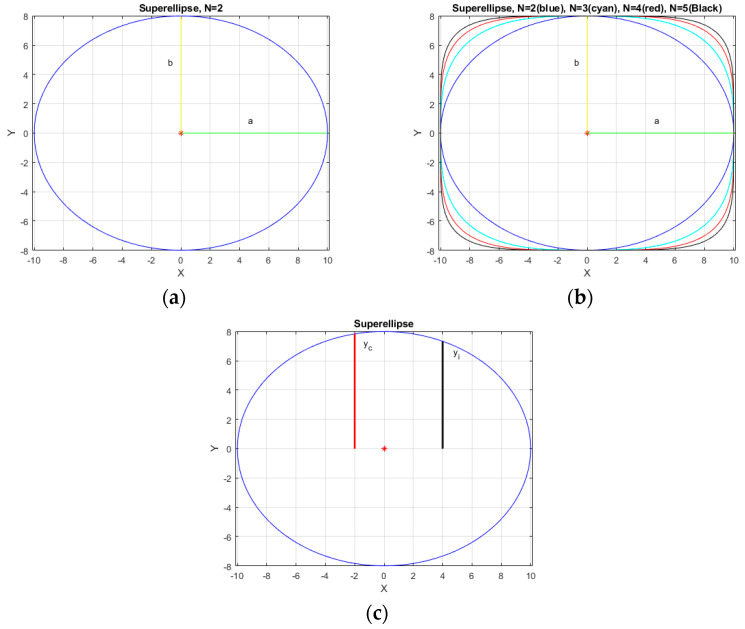
Superellipse with (**a**) N = 2 (**b**) N = 2 (blue), 3 (cyan), 4 (red), 5 (black). The curve is changed to more cuboidal when N increases. (**c**) The superellipse modification in our 3D reconstruction model.

**Figure 3:**
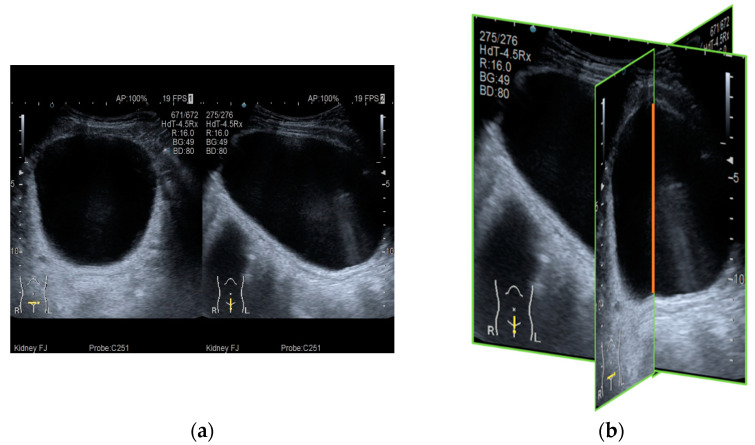
(**a**) Original 2D ultrasonic image. (**b**) The transverse view and sagittal view are aligned (crossover) in the maximum vertical line.

**Figure 4:**
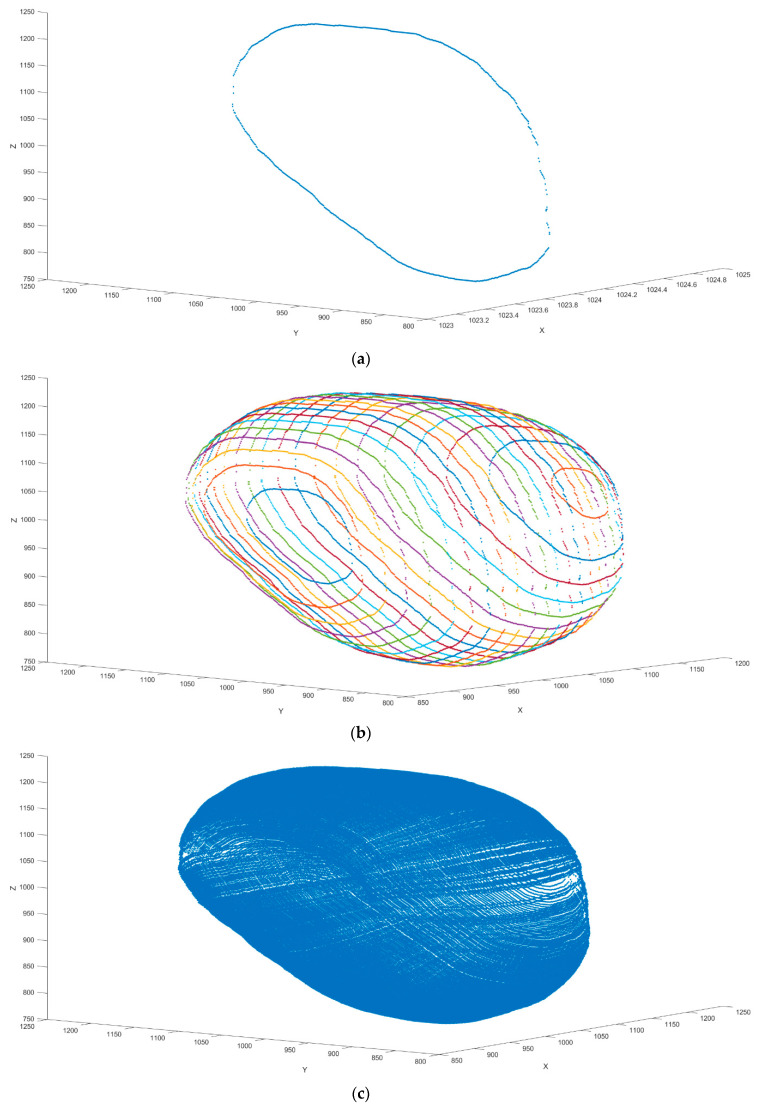
(**a**) Original sagittal view of bladder. (**b**) Generate new sagittal image through our 3D reconstruction equation. (**c**) Multiple new sagittal images stack into 3D volume.

It can be seen that when N changed in the equation, the shape of the superellipse also changed (Figure 2). Our bladder volume 3D reconstruction model is established from transverse view and sagittal view of general bladder ultrasonic images. We aligned the transverse view and sagittal view in the maximum vertical line (Figure 3). Due to the pelvic bone around the bladder, the bladder shape is restricted in a confined space inside the pelvis. We used the superellipse volume reconstruction as a 3D bladder reconstruction model to simulate the edge reduction near the pelvic cavity wall. According to the superellipse shape modified in each pixel of the width of the transverse view, we generated several new sagittal images ($S_{i}$) from the original crossover sagittal view ($S_{c}$). Each new generated sagittal view stacks and builds the 3D bladder volume model (Figure 4). The formula of bladder volume 3D reconstruction model is listed as follows:

(3) $$3D\;Reconstruction\;Volume=Multiple\;New\;Sagittal\;Images\;\left(S_{i}\right)\;stacks\;=\overset{a}{\underset{i=-a}{\sum }}S_{i}=\overset{a}{\underset{i=-a}{\sum }}S_{c}\times \frac{\left|\frac{y_{i}}{b}\right|}{\left|\frac{y_{c}}{b}\right|}\;=\overset{a}{\underset{i=-a}{\sum }}S_{c}\times \frac{\sqrt[{n}]{1-{\left|\frac{x_{i}}{a}\right|}^{n}}}{\sqrt[{n}]{1-{\left|\frac{x_{c}}{a}\right|}^{n}}}\;-a<x_{i}<a\;;\;\;a=\frac{Width\;in\;Transverse\;View}{2}\;;\;\;c=crossover\;point$$

We used this new 3D reconstruction model with different N values (N = 2, 3, 4, 5) in the superellipse modified (Figure 2C). With a higher value of N in the superellipse equation, the shape is more similar to the rectangle, which is compatible with intrapelvic space. We used different N values to find the best accuracy in this model. There are 11 pairs of images from the pre-voiding volume and post-voiding volume in people with incomplete bladder emptying. In this situation, we compared the actual volume with the difference between the pre-voiding estimated volume and the post-voiding estimated volume in both the traditional bladder volume measurement method and our 3D bladder reconstruction model. Results from the traditional method and our novel 3D bladder volume reconstruction model were statistically analyzed. We also classified the results into four different groups of volume (i.e., 0–200 mL, 200–400 mL, 400–600 mL, >600 mL) and performed further analysis. Student’s t-test and the Wilcoxon signed-rank test were performed for statistical analyses. Scatterplots of correlation and the Bland–Altman plot were used to compare the results. All model calculations and statistical analyses were performed using software, i.e., MATLAB version 2017b (The MathWorks, Inc., Natick, MA, USA).

## 3. Results

A total of 75 paired B-mode bladder ultrasound images and 64 actual urine volume records were collected. There were 19 female and 45 male patients with a mean age of 64.5 years (range, 24 to 92) (Table 1). The mean actual volume is 531.8 mL (range of 101.9–1400 mL). The mean estimated volume is 385.5 mL (range of 48.5–887.7 mL) in the traditional method. In our 3D reconstruction model, the mean estimated volume is 467.5 mL (range of 96.3–1186 mL) when N = 2; 503.9 mL (range of 109.8–1339.4 mL) when N = 3; 529.9 mL (range of 102.9–1451.7 mL) when N = 4; and 546.6 mL (range of 100.9–1514.2 mL) when N = 5. The mean percentage error of the traditional estimation method is −28% ± 10.15% (mean ± standard deviation). In our 3D reconstruction model, the mean error is −10.18% ± 20.69% when N = 2, −4.72% ± 7.38% when N = 3, −0.33% ± 3.86% when N = 4, and 2.58% ± 3.65% when N = 5. The statistical difference was analyzed between the actual volume and estimated volume. Only when N = 4 in the 3D reconstruction estimated model was there no significant difference with the actual volume in Student’s t-test and the Wilcoxon signed-rank test (Table 2).

**Figure 5:**
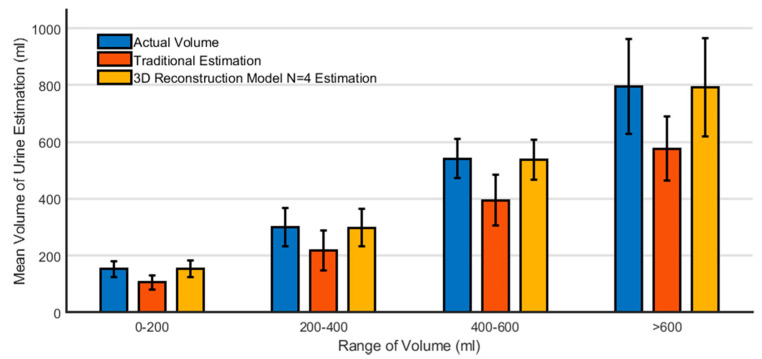
Actual volume compared with traditional and 3D reconstruction estimated volume in four different groups of volume (0–200, 200–400, 400–600, >600 mL).

**Figure 6:**
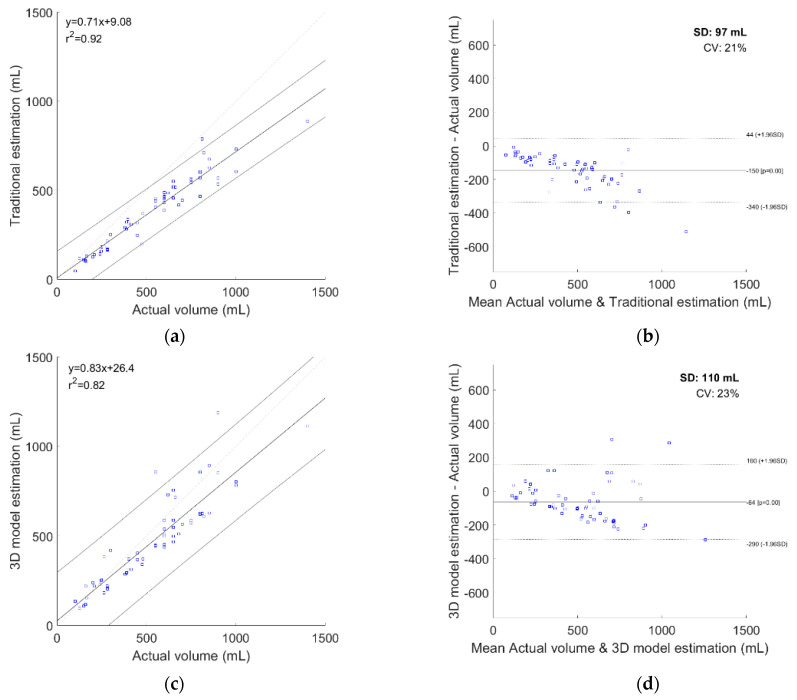

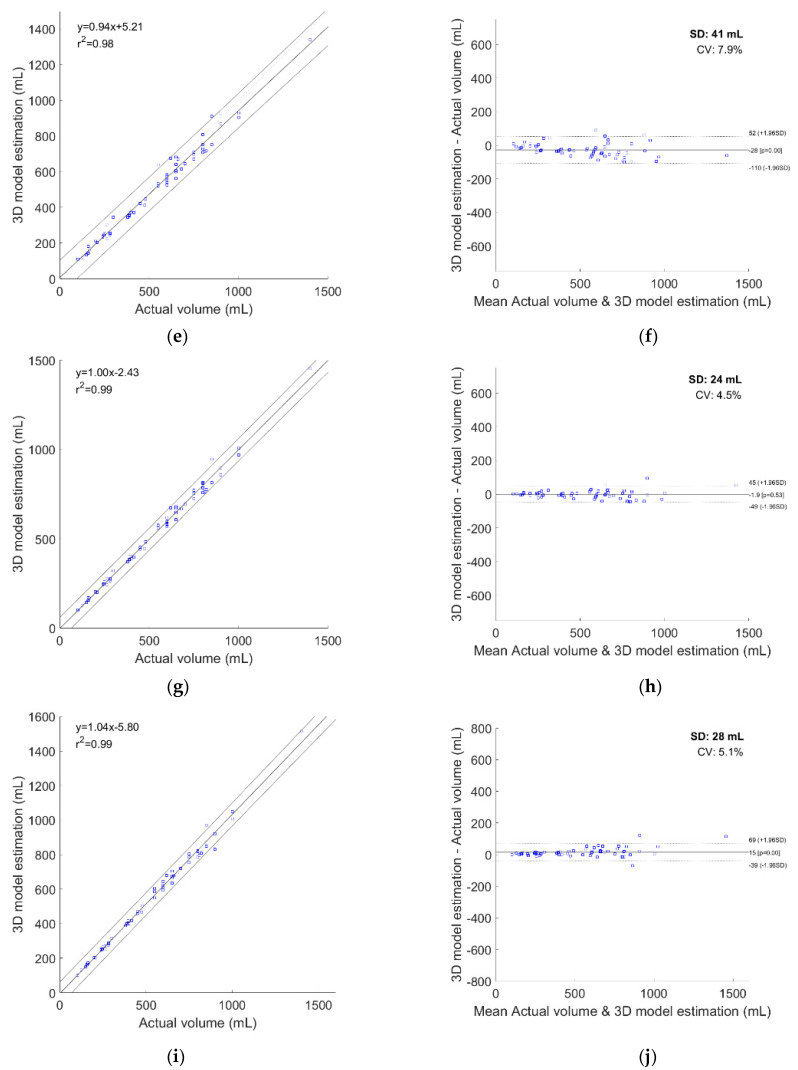
Traditional and 3D reconstruction model estimation result. (**a**) Correlation with 95% confidence interval and linear regression equation in Traditional estimation. (**b**) Difference in the Bland–Altman plot with 95% confidence interval in Traditional estimation. (**c**) Correlation with 95% confidence interval and linear regression equation in 3D reconstruction of N = 2 model. (**d**) Difference in the Bland–Altman plot with 95% confidence interval in 3D reconstruction of N = 2 model. (**e**) Correlation with 95% confidence interval and linear regression equation in 3D reconstruction of N = 3 model. (**f**) Difference in the Bland–Altman plot with 95% confidence interval in 3D reconstruction of N = 3 model. (**g**) Correlation with 95% confidence interval and linear regression equation in 3D reconstruction of N = 4 model. (**h**) Difference in the Bland–Altman plot with 95% confidence interval in 3D reconstruction of N = 4 model. (**i**) Correlation with 95% confidence interval and linear regression equation in 3D reconstruction of N = 5 model. (**j**) Difference in the Bland–Altman plot with 95% confidence interval in 3D reconstruction of N = 5 model. SD: standard deviation; CV: coefficient of variation.

After the data were divided into four different groups of volume, there are significant differences found between the 3D reconstruction N = 4 model estimation and the traditional estimation in all four groups (Table 3). There is no significant difference in Student’s t-test and the Wilcoxon signed-rank test between the actual volume and the 3D reconstruction N = 4 model estimation in each group (Figure 5). High correlations were found between the actual bladder volume and all estimated methods. Pearson’s correlation coefficient is 0.96 (R2 = 0.92) in the traditional estimation; 0.91 (R2 = 0.82) in the 3D reconstruction model with N = 2; 0.989 (R2 = 0.98) when N = 3; 0.996 (R2 = 0.992) when N = 4; and 0.9958 (R2 = 0.9916) when N = 5. Standard deviation with the actual volume is 97 mL (coefficient of variation = 21%) using the traditional estimation; 110 mL (coefficient of variation = 23%) in the 3D reconstruction model with N = 2; 41 mL (coefficient of variation = 7.9%) when N = 3; 24 mL (coefficient of variation = 4.5%) when N = 4; 28 mL (coefficient of variation = 5.1%) when N = 5. The correlation scatterplot of the actual volume against the estimated volume is shown in Figure 6a, and a linear regression with 95% confidence interval is also presented. The Bland–Altman plot was performed to show the differences in each estimated method (Figure 6b) with 95% confidence interval. The difference decreased in our 3D reconstruction model when N increased gradually, and the best accuracy was found when N = 4. After N more than four in the 3D reconstruction model, the error rate increased gradually.

## 4. Discussion

In lower urinary tract disease, post-voided residual urine in the bladder is very important in the decision making of the diagnosis. Urinary catheterization is the most accurate method in determining the actual residual urine in the bladder, but it is also an invasive procedure that causes patients more discomfort and infection. Catheterization-associated urinary tract infection is also one of the most common healthcare-associated infections [16]. Bladder sonography is a non-invasive study for estimating bladder volume, first performed in 1967 [2]. Bladder sonography was recommended as a replacement for catheterization in measuring bladder residual urine by Roehrborn and Peters in 1988 [14]. However, the residual urine predicted from bladder sonography is not as accurate as it is from urinary catheterization. Three-dimensional ultrasound for bladder volume estimation is developing and has a better correlation coefficient of 0.92–0.94 between the actual volume and estimated volume [5].

Post-voided residual urine measurement is recommended for all patients with urinary incontinence by the Agency for Health Care Policy and Research in the United States [17]. Urinary incontinence can be briefly classified into three types: urge urinary incontinence (UUI), stress urinary incontinence (SUI), and overflow urinary incontinence. Stress urinary incontinence occurs when urine leaks with increasing abdominal pressure, such as when laughing, coughing, and exercise. Urge urinary incontinence and overflow urinary incontinence have mimicked symptoms. Overflow incontinence is a condition of urine retention and causes severe frequency with a small amount of urinary incontinence when the bladder receives external pressure from the abdomen. The amount of bladder residual urine volume is the key point in distinguishing between urge urinary incontinence and overflow incontinence. The accuracy of bladder residual urine estimation is essential in the clinical judgment of urine retention, especially when facing a confused moderate amount in volume estimation. Increase accuracy without high costs is helpful in diagnosis and makes it more accessible with clinical practice.

Bladder outlet obstruction can also cause difficulty voiding, even urine retention. Bladder outlet obstruction may have originated in pelvic organ prolapse, prostate enlargement, and urethral lesions such as urethral stenosis or urethral caruncle. Pelvic organ prolapse produces external compression on the urethra or bladder neck, leading to a weaker urine stream. Urethral lesions such as urethral stenosis or urethral caruncle decrease the urethral lumen area and cause difficulty voiding. In some male patients without prostate enlargement and bladder outlet obstruction, they still have lower urinary tract symptom and receive Botulinum toxin injection on the bladder neck and urethra to improve the symptom. Bladder capacity and post-void residual volume are also evaluated as a marker of improvement [18]. The measure of bladder residual urine volume is the basis for judging the severity of bladder outlet obstruction and influencing the treatment given to the patient.

The variation of pelvic architecture could influence the bladder volume with fullness. There are four main types of the pelvis in humans, namely the gynecoid, android, anthropoid, and platypelloid types; the gynecoid type is round with a wide-open outlet, which is the most common type (about 51.3%) in females for vaginal delivery [19]. The android type of pelvis is narrow with heart-shape, which is found in most of the males or females with more physical activity in the adolescence period [20]. A great variation of the bladder volume in the infant (less than one year old) is also found between the estimation from the current formula and actual volume [21]. Further study with more samples may emphasize the factors that could affect the bladder volume, and accuracy may further increase under different factors.

Demaria et al. found that using a commercial bladder scanner with a special 3D ultrasound probe is accurate for estimating immediate postpartum urine retention volume in women who delivered vaginally [22]. Using a commercial bladder scanner is not economical due to the high cost, and it only has a single function, i.e., to check the volume of bladder residual urine [23]. This equipment is destined to be set up only in special departments, such as obstetrics and gynecology or urology. It is not possible for the device to see popular use in the general ward or in general clinics due to restricted application and high costs. In recent years, several portable small convex ultrasound probes were invented, enabling easy connection with a general notebook or a mobile phone [24,25]. With this approach, the probes transfer general B-mode (2D) ultrasound signals into the general device.

In our study, this conventional bladder sonography process into three-dimensional modeling volume calculation provides more accuracy than the traditional measurement method. The error decreases when the model N increases. Furthermore, this 3D bladder reconstruction model can achieve no statistically significant difference from the actual volume when N = 4. In previous studies, the bladder volume measurements were less accurate when the volume was too small or too big [26]. We divided the data into four groups according to the different volume levels and found that the same trend can be observed across all four groups (Figure 5). In other words, this new bladder measurement method is reliable even in small or large volume estimation.

This whole process can be built as an automatic process in notebook software or mobile phone apps. In this way, the 2D signals from the small portable convex ultrasound probe can be sent as input to the software or app, and the automatic volume calculation of bladder residual urine can be computed. It provides a low-cost solution to general departments that need to easily obtain information on residual bladder urine volume through general B-mode (2D) ultrasound. The general B-mode ultrasound also has wider application in other clinical practice than restricted commercial bladder scanners. This novel 3D bladder reconstruction model for volume measurement provides the possibility of low-cost application and wider usage for other clinical members.

## Figures and Tables

**Table 1:** Patient characteristics.

Characteristics	No. of Patients
Age (yr) ^1^	64.53 ± 14.24 (24–92)
Gender	
Male	45
Female	19
Total	64
Actual bladder volume (mL)	
0–200	8
200–400	16
400–600	15
>600	25

^1^ Age: mean ± standard deviation (range).

**Table 2:** Estimated bladder volume and percentage error.

	Volume (mL) ^1^	Mean Percentage Error (%) ^2^	Mean Absolute Percentage Error (%)	Student’s *t*-Test, *p*-Value ^3^	Wilcoxon Signed-Rank Test, *p*-Value ^3^
Actual volume	531.8 ± 268.7 (101.9–1400)				
Traditional Estimation	385.5 ± 198.5 (48.5–887.7)	−28.00 ± 10.15	28.00 ± 10.15	*p* < 0.001	*p* < 0.001
3D reconstruction model (N = 2)	467.5 ± 245.5 (96.3–1186.0)	−10.18 ± 20.69	20.48 ± 10.37	*p* < 0.001	*p* < 0.001
3D reconstruction model (N = 3)	503.9 ± 254.6 (109.8–1339.4)	−4.72 ± 7.38	7.79 ± 3.94	*p* < 0.001	*p* < 0.001
3D reconstruction model (N = 4)	529.9 ± 270.0 (102.9–1451.7)	−0.33 ± 3.86	3.08 ± 2.33	0.49 ^4^	0.3062 ^5^
3D reconstruction model (N = 5)	546.6 ± 280.2 (100.9–1514.2)	2.58 ± 3.65	3.54 ± 2.71	*p* < 0.001	*p* < 0.001

^1^ Mean ± standard deviation (range). ^2^ Mean ± standard deviation. ^3^ Comparation of differences with actual volume. ^4^ Statistically no difference between actual volume and 3D reconstruction model (N = 4) in Student’s *t*-test. ^5^ Statistically no difference between actual volume and 3D reconstruction model (N = 4) in the Wilcoxon signed-rank test.

**Table 3:** Estimated results in different groups.

	Actual Volume	Traditional Estimation			3D Reconstruction Model Estimation (N = 4)			*p*-Value ^1^
		Volume (mL)	Mean Percentage Error (%)	Mean Absolute Percentage Error (%)	Volume (mL)	Mean Percentage Error (%)	Mean Absolute Percentage Error (%)	
0–200	152.5 ± 28.9	106.0 ± 25.4	−30.6 ± 13.8	30.6 ± 13.8	153.2 ± 30.0	0.44 ± 2.97	2.45 ± 1.50	<0.001
200–400	300.4 ± 66.3	218.2 ± 71.0	−28.7 ± 9.1	28.7 ± 9.1	298.7 ± 66.0	−0.55 ± 4.10	3.20 ± 2.50	<0.001
400–600	541.4 ± 61.1	394.8 ± 89.1	−27.7 ± 11.0	27.7 ± 11.0	538.1 ± 71.2	−0.65 ± 3.36	2.78 ± 1.87	<0.001
>600	795.6 ± 166.0	576.4 ± 112.6	−26.9 ± 9.4	26.9 ± 9.4	793.5 ± 173.2	−0.26 ± 4.38	3.39 ± 2.70	<0.001
Total	531.8 ± 268.7	385.5 ± 198.5	−28.0 ± 10.1	28.0 ± 10.1	529.9 ± 270.0	−0.33 ± 3.86	3.08 ± 2.33	<0.001

^1^*p*-value < 0.001 is the significant difference between traditional estimation and 3D reconstruction model estimation (N = 4) in both Student’s *t*-test and the Wilcoxon signed-rank test.

## Data Availability

Not applicable.
